# Global Neurocognitive and Emotional Dysfunction in Fanconi Anemia: A Neuropsychological Case Report of a 39-Year-Old Patient

**DOI:** 10.1155/crnm/6424739

**Published:** 2025-09-30

**Authors:** Ollie Fegter, Brian M. Cerny, Jason R. Soble

**Affiliations:** ^1^Department of Psychiatry, University of Illinois Chicago College of Medicine, Chicago, Illinois, USA; ^2^Department of Psychiatry and Behavioral Sciences, Northwestern University Feinberg School of Medicine, Chicago, Illinois, USA; ^3^Department of Neurology and Rehabilitation, University of Illinois Chicago College of Medicine, Chicago, Illinois, USA

**Keywords:** case report, cognitive impairment, Fanconi anemia, neuropsychology

## Abstract

Fanconi anemia is a rare genetic disorder characterized by impaired DNA repair, leading to bone marrow failure, congenital anomalies, and increased cancer risk. Intellectual disability and developmental disorders in Fanconi anemia have been briefly described in previous studies, but there has been limited in-depth examination of cognitive functioning associated with Fanconi anemia. This case report presents comprehensive neuropsychological findings from a 39-year-old woman with Fanconi anemia, detailing significant cognitive impairment, including intellectual disability with concomitant deficits in learning and memory, processing speed, and complex attention/executive functions, while basic language and basic visuospatial skills remained relatively preserved. Neuroimaging revealed nonspecific cerebral calcifications. This cognitive profile aligns with limited existing reports suggesting intellectual disability and global cognitive impairment in Fanconi anemia. This case highlights the critical gap in Fanconi anemia literature regarding comprehensive neuropsychological data and underscores the necessity of systematic cognitive assessments in this population. Future research should include large-scale and longitudinal studies, potentially incorporating standardized cognitive testing within existing frameworks such as the International Fanconi Anemia Registry, to better characterize and understand cognitive trajectories and to develop targeted interventions to enhance the quality of life in individuals with Fanconi anemia.

## 1. Introduction

Fanconi anemia (FA) is a rare genetic disorder characterized by defects in DNA repair mechanisms and predominantly manifests through bone marrow failure, congenital abnormalities, and an increased predisposition to leukemia and solid tumors [1–6]. Diagnosis typically occurs in childhood or young adulthood [7]. Patients often present with a combination of physical, neurologic, and cognitive/psychiatric symptoms. Physical symptoms include short stature, skeletal deformities (including thumb and radial anomalies), microcephaly, hyperpigmentation, gonadal dysfunction leading to infertility and early menopause, hearing loss, ocular malformations, and cardiac and renal abnormalities [1, 8–11]. Neurologic presentation can include seizures, ataxia, visual disturbances, spasticity, paresthesia, and stroke-like symptoms [12, 13].

FA exhibits variable prevalence across different populations, with an overall incidence of approximately 1 in 136,000 live births globally [4, 14]. However, certain populations demonstrate significantly higher rates due to founder mutations, including individuals of Ashkenazi Jewish (carrier frequency ∼1 in 90–100), Afrikaner (∼1 in 83), Spanish Romani (∼1 in 64–70), and sub-Saharan African (∼1 in 40,000 births) descent [15]. Consanguinity further concentrates disease prevalence in some communities.

Up to 90% of patients with FA can have abnormal neuroimaging findings, including corpus callosum abnormalities, small pituitary glands, cerebellar lesions, and posterior fossa lesions [12, 13, 16, 17]. Specifically, imaging findings may include cerebral and cerebellar lesions with calcifications and ring-enhancing lesions [12]. The proposed mechanisms underlying these abnormalities are likely multifactorial. Research in mice has shown that neural stem cell depletion may play a central role, with FA DNA repair pathways leading to decreased neural progenitor cell proliferation, increased neural stem cell exhaustion with aging, and reduced self-renewal capacity in neural stem cells [18]. Simply put, congenital DNA damage may drive premature stem cell aging [16]. Observations in pediatric populations have suggested that acquired abnormalities, such as white matter T2 hyperintensities and vascular lesions, may result from DNA repair mechanisms leading to gliosis [16]. More recently, one group described Fanconi anemia neuroinflammatory syndrome (FANS), characterized by progressive neurologic symptoms and brain lesions with calcifications, wherein histological analysis revealed polyomavirus-infected microglial cells, suggesting viral and inflammatory contributions to neurological manifestations [12].

Cognitive and psychiatric symptoms are also common and may include speech impairment, mood and adjustment disorders, developmental delays and intellectual disabilities, and in one published case report, psychosis [11, 19–22]. Unsurprisingly, individuals with significant cognitive and psychiatric symptoms may experience educational, occupational, and interpersonal difficulties [20, 21, 23].

Despite these reports, comprehensive neuropsychological data in FA are scarce. The available literature typically provides brief mentions of developmental delays or intellectual disabilities without comprehensive neurocognitive data [16, 19, 22]. To date, only one study [11] has presented extensive neuropsychological findings, in a case report of a 17-year-old FA patient presenting with psychotic symptoms and intracranial calcification following irradiation and bone marrow transplant. They documented significant global impairments across cognitive domains, including executive functions, memory, attention, visuospatial processing, and verbal comprehension, with notable cognitive decline observed over a two-year period.

Given the paucity of comprehensive neuropsychological studies, no established neuropsychological profile exists for FA. Consequently, the literature on the extent, nature, and pattern of neurocognitive and behavioral impairment in FA remains largely undeveloped. The purpose of this paper is to begin to address this gap by presenting a detailed case report from a neuropsychological evaluation of an adult with FA. This work aims to contribute to the establishment of a clearer neuropsychological profile for individuals with FA and to inform future clinical and research practices.

## 2. Case Presentation

The patient is a 39-year-old, right-handed, Black American woman. She presented to an outpatient neuropsychology clinic housed in an urban midwestern academic medical center upon referral from her neurology provider and was accompanied by her mother. English is her only language.

The patient's birth and early development aside from her FA diagnosis were largely unremarkable. Her mother reported that as a child, the patient had difficulty paying attention and finishing projects, memory problems, poor self-esteem, was easily frustrated, depressed, impulsive, and had poor coordination. The patient's mother reported the patient had always acted immature for her age. In school, she was a below average student and had difficulty with math, reading, and social studies. She was diagnosed with an unspecified learning disorder and had an individualized education plan (IEP), which included tutoring and formal classroom accommodations. She completed an associate degree at a community college in mainstream classes with read-aloud accommodations for written material on assignments and exams. Occupationally, she has assisted family members in daycare and grocery settings but has never held competitive employment. She has never been married and does not have children.

Relevant medical history was remarkable for FA, hypertension, liver steatosis, asthma, gastroesophageal reflux disease (GERD), and tension-type headaches. She denied history of seizure, stroke, heart attack, or surgeries. Additionally, she denied history of head injury with loss of consciousness or alterations in mental status. Psychiatric history was remarkable for anxiety, depression, panic attacks, alcohol use disorder, and attention-deficit/hyperactivity disorder (ADHD; diagnosed in adulthood). Psychiatric treatment history included medication management, individual psychotherapy, and group therapy at a community mental health center. Notably, she denied improvement in cognitive complaints but described acute exacerbation of compulsive behaviors with prescribed amphetamine salts (i.e., purchasing, gambling, collecting, and counting). Prescribed medications included albuterol, amlodipine, amphetamine-dextroamphetamine, baclofen, duloxetine, fluticasone, hydrocodone-acetaminophen, hydrocortisone-pramoxine foam, ibuprofen, lidocaine patch, norethindrone, omeprazole, and oxybutynin. The patient confirmed she was not taking amphetamine-dextroamphetamine on the day of testing but was unable to provide additional information regarding medication doses, indications, or adherence. This information was not available in accessible medical records. Family medical history was remarkable for FA and autism spectrum disorder in her brother, lung cancer and polymyositis in her father, heart attack in both maternal grandparents, ADHD and unspecified learning disorders in her mother and brothers, and anxiety in her mother.

**Figure 1:**
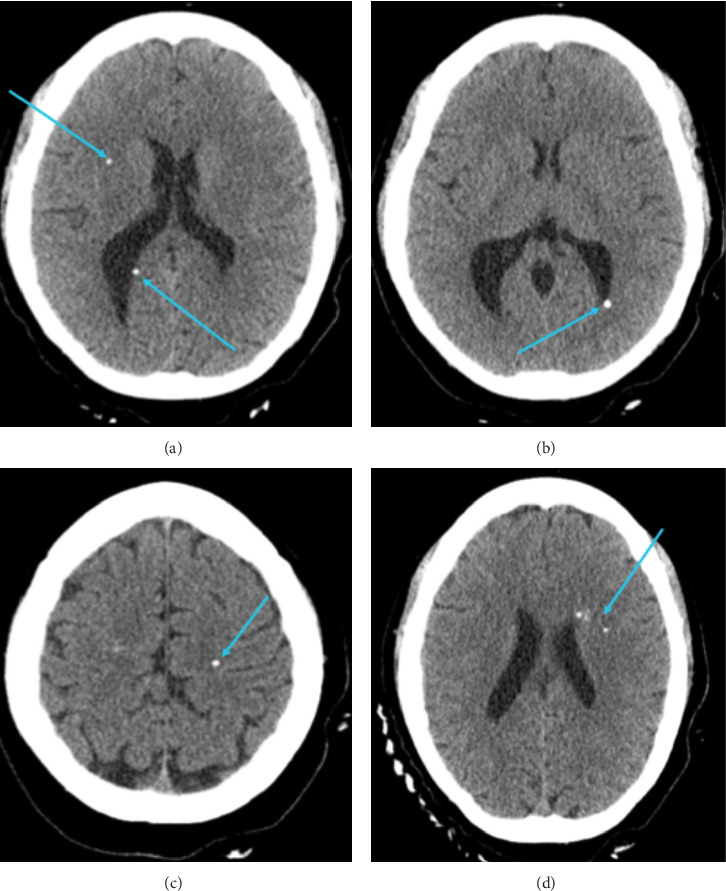
CT scan of the patient's brain with calcified lesions identified. Axial CT images without contrast demonstrating multiple calcifications throughout the brain. (a)–(d) show representative axial slices, with blue arrows indicating dense calcifications scattered throughout the bilateral cerebral hemispheres and brainstem. No acute intracranial abnormality, mass effect, or midline shift was identified.

**Figure 2:**
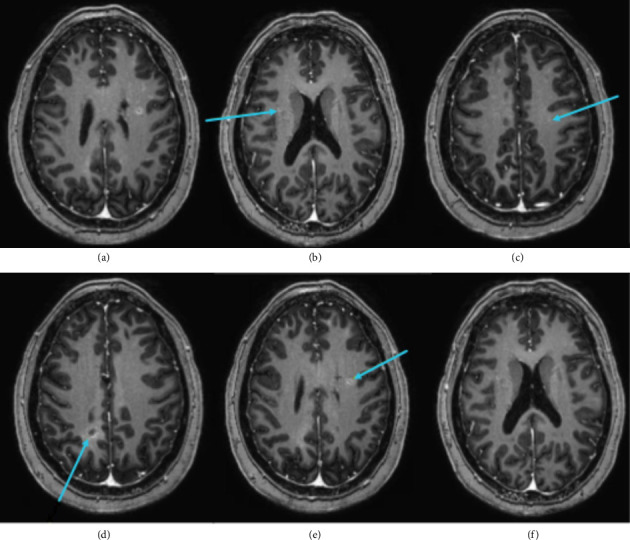
MRI scan of the patient's brain with calcified lesions identified. Axial MRI images showing multiple scattered punctate areas of susceptibility artifact corresponding to calcifications seen on CT. (a)–(f) show representative axial slices, with blue arrows indicating susceptibility artifacts throughout the bilateral cerebral hemispheres and brainstem. These findings were interpreted as most likely representing neurocysticercosis, though infectious disease workup was unremarkable. Additionally, two areas of contrast enhancement near the occipital horn of the right lateral ventricle represented developmental venous anomalies.

The patient was referred to neuropsychology due to cognitive complaints and abnormal findings on neuroimaging. A computed tomographic (CT) scan was conducted after the patient fell and hit her head, approximately 1 year prior to neuropsychological evaluation. This fall did not result in altered mental status or loss of consciousness. The CT scan (see Figure 1) showed no acute intracranial abnormality, but with nonspecific calcifications within the bilateral cerebral hemispheres and brainstem. Subsequently, a magnetic resonance imaging (MRI) scan was performed approximately 4 months prior to neuropsychological evaluation (see Figure 2). MRI showed multiple scattered punctate areas of susceptibility artifact throughout the bilateral cerebral hemispheres and brainstem, corresponding to calcifications previously seen on CT. More specifically, CT imaging revealed dense calcifications within the pons and scattered calcifications throughout the bilateral cerebral hemispheres, while MRI demonstrated preserved normal gray-white matter differentiation, intact cortical sulci, and normal ventricular morphology. The calcifications appeared as punctate susceptibility artifacts distributed diffusely across both hemispheres, with some lesions showing thin rims of surrounding contrast enhancement suggestive of perilesional edema. Notably, imaging reports listed neurocysticercosis as a potential etiology for cerebral calcifications, but infectious disease workup was unremarkable, and the patient had not traveled outside of the United States. Additionally, there were two areas of contrast blushing seen superiorly inferior to the occipital horn of the right lateral ventricle, representing developmental venous anomalies.

On evaluation, the patient reported lifelong problems with short-term memory exacerbated by stress but denied any other cognitive concerns. Her mother reported that the patient had lifelong problems with memory, concentration, word finding, planning and organizing, getting lost, episodes of confusion, and difficulty thinking clearly and efficiently, which had worsened over the past year. The patient complained of chronic daily headaches, lower extremity weakness, and frequent falls over the past year. Her mother reported that the patient experienced lifelong fatigue, as well as more recent shakiness, and expressed that the patient's falls occurred largely in the context of alcohol intoxication. The patient and her mother denied that the patient had experienced acute sensory changes, gait disturbance, hallucinations, incontinence, fluctuating mental status, or other chronic pain. The patient reported her sleep is poor; she has no consistent sleep schedule and has difficulty with sleep initiation and maintenance. She frequently stays up late watching television or communicating with friends. She denied snoring or disruptive sleep behaviors.

Psychologically, the patient reported significant anxiety and depression, related to family stressors and discord, loneliness, and lack of independence. She described lifelong difficulty maintaining quality friendships and evidenced limited insight into relationships and social pragmatics. Her mother described the patient as having significant emotional lability, poor coping/emotion regulation skills, and immature behavior for her age. The patient and her mother reported that the patient had lifelong compulsive buying and hoarding behaviors of “anything on sale,” including collectible cups, trinkets, and clothing. Additionally, they reported the patient engaged in compulsive daily gambling via smartphone applications. The patient currently drinks alcohol about 4 days per week and typically has 5 or more drinks at a time. She reported heavier past use, which caused problems at home with family and at work. She smokes cigarettes socially—about one pack per week—and occasionally uses cannabis.

At the time of evaluation, the patient lived in a house with her mother and brother. She was independent in basic activities of daily living, but dependent on her mother for all instrumental activities of daily living including managing medical appointments, medications, and finances. The patient was previously driving, but more recently prohibited by her mother after she received a legal citation for driving under the influence (DUI). The patient did not know how to use public transportation, had never held competitive employment, and had significant credit card debt secondary to compulsive purchasing and gambling.

On the day of evaluation, the patient presented casually dressed and adequately groomed. She appeared mildly microcephalic and younger than her stated age. She was alert, oriented, and cooperative throughout testing, with adequate vision and hearing. She demonstrated generally organized, logical, and goal-directed thought processes, with no evidence of psychosis. Mood was variable with notable emotional dysregulation, including episodes of tearfulness. Significant difficulties were evident in abstract reasoning, perspective-taking/theory of mind, and insight. Social comportment and pragmatics were reduced, and she showed difficulty with conversational reciprocity. She demonstrated low frustration tolerance and required frequent encouragement and repetition of test instructions. Functional autobiographical memory and orientation were grossly intact. She explicitly denied suicidal or homicidal ideation, plans, or intent and was not in acute crisis during the evaluation.

Neuropsychological tests administered as part of the evaluation are listed in Table 1, and the patient's test performance is shown in Table 2. The neuropsychological battery was designed to comprehensively sample across major cognitive domains following standard assessment guidelines [24]. Based on prior literature suggesting developmental delays and intellectual disabilities in FA, we anticipated the possibility of global cognitive impairment and selected measures to characterize functioning across multiple domains. As part of the evaluation, the patient was administered 5 freestanding performance validity tests (PVTs) and 4 embedded PVTs; she performed within acceptable limits on 4/5 freestanding and 4/4 embedded PVTs, indicating her test data were valid for clinical interpretation [25].

Her neuropsychological profile was remarkable for extremely low general cognitive/intellectual functioning with concomitant deficits in learning/memory (nonamnestic), processing speed, and complex attention/executive functions. Neurobehaviorally, she demonstrated severe emotional dysregulation, compulsive behaviors (hoarding, purchasing, and gambling), reduced insight, poor social reciprocity, and excessive alcohol consumption. In contrast, basic attention, language, and visuoconstructional abilities remained functional. Motor testing revealed bilaterally impaired strength and speed/dexterity. Health numeracy and health literacy were both reduced [55–57]. Psychologically, she reported severe symptoms of anxiety [58] and depression [59] in the context of moderate childhood emotional abuse and neglect, as well as low-moderate physical abuse and neglect, and sexual abuse [60]. On an observer/collateral report measure of adaptive functioning, she demonstrated severely impaired adaptive skills and dependence on her mother for all instrumental activities of daily living [61].

As such, this patient's data and history were primarily consistent with an intellectual developmental disorder, likely secondary to FA. It was noted that the neurocognitive deficits observed on testing also may have represented a decline from her premorbid baseline secondary to alcohol abuse, significant mood distress with medication noncompliance, sleep disturbance, and cerebral calcifications seen on imaging, but the extent of any such decline was difficult to establish given premorbid intellectual disability and lack of prior neuropsychological assessment. This patient was diagnosed with a mild-to-moderate intellectual developmental disorder, alcohol use disorder, and unspecified anxiety and depressive disorders.

## 3. Discussion

This is the first report to provide a comprehensive neuropsychological profile of an adult patient with FA. This case addresses a critical void in the FA literature by thoroughly characterizing neurocognitive function, offering novel insights into a neuropsychological phenotype of FA. The patient's neuropsychological profile was characterized by low general intellectual functioning, and pronounced weaknesses on tasks requiring sustained mental efficiency, working memory, and higher-order planning and organization, alongside relatively preserved basic language and visuospatial abilities. In the context of low general intellectual functioning, this pattern of widespread cognitive impairment affecting complex cognitive processes while preserving basic language and visuospatial skills is consistent with an underlying neurodevelopmental disorder impacting multiple brain systems, as opposed to focal dysfunction.

The specific pattern of neuroimaging abnormalities may explain the pattern of strengths and weaknesses observed on testing. The scattered punctate calcifications throughout the bilateral cerebral hemispheres occurred within the context of relatively preserved cortical architecture and normal gray-white matter differentiation. This may explain why basic functions such as vocabulary knowledge—mediated by the left inferior frontal gyrus and anterior temporal lobe—and simple visuoconstruction—mediated by the right inferior parietal lobe—remained relatively intact while complex cognitive processes reliant on distributed neural networks showed significant impairment. The bilateral and diffuse nature of her hemispheric calcifications may have disrupted white matter connectivity and within frontoparietal networks necessary for complex learning and memory consolidation, working memory, and other executive functions [62]. Additionally, the patient's severe motor impairments on both grip strength and fine motor dexterity tasks may reflect disruption of motor networks with cerebellar connections, given brainstem involvement.

The cognitive effects of this patient's comorbid conditions warrant consideration. Alcohol use disorder typically produces impairments in executive functioning, working memory, processing speed, and learning, though this patient showed no evidence of Wernicke's encephalopathy or Wernicke–Korsakoff syndrome [63]. Depression and anxiety commonly affect processing speed, working memory, and memory retrieval [64, 65]. Sleep disturbance may impact sustained attention, processing speed, working memory, and executive functions [66, 67]. Notably, there is substantial overlap between these expected patterns and those observed in our patient. However, certain features of her profile may be more specifically attributable to FA, including the severity of her intellectual disability, extensive motor impairments, and visual-motor integration deficits observed in intellectual developmental disorders [68]. Additionally, the observed cerebral calcifications provide a neurobiological substrate that may account for cognitive impairment independent of psychiatric and substance-related factors.

These findings provide further, detailed support for prior observations of cognitive impairment in FA, even though such reports have been limited. Earlier studies and case descriptions have noted that some individuals with FA experience mild intellectual disability or developmental delays from childhood [16, 19, 22]. Similar to the only known FA case report presenting neuropsychological data, we found a wide range of cognitive impairment across cognitive domains and multiple cerebral calcifications [11]. Interestingly, this patient did not have history of focused or whole-body irradiation, cancer, or FA-related tumors, unlike the patient described by Iki et al. [11]. The presented patient's profile reinforces that cognitive deficits, particularly intellectual developmental disorders, and idiopathic cerebral calcifications may represent a distinct phenotype of FA. Importantly, in both the current patient and the previously reported case, there is a common thread of global cognitive vulnerability leading to academic and occupational difficulties that necessitate special education and workplace accommodations, as well as specialized neuropsychiatric care.

It is worth noting that similar patterns of cognitive impairment have been reported in other rare genetic disorders that, like FA, involve DNA repair deficits or bone marrow failure. For example, ataxia-telangiectasia (AT) is a DNA-repair disorder that often features progressive neurological decline [69–71]. One study found that in pediatric patients, even in early AT stages, cognitive impairment was present, including lower performance in overall intelligence, processing speed, working memory, and executive tasks when compared to peers [72]. With disease progression, they found further decline in attention and abstract reasoning, indicating broad cognitive involvement [72]. Another example is xeroderma pigmentosum (XP), a genetic disorder characterized by a decreased ability to repair DNA damage, such as that caused by ultraviolet light exposure [73–75]. Specifically in its neurological forms, individuals with XP may exhibit developmental delays to moderate to severe intellectual disabilities, with common cognitive impairments in attention, executive function, and memory [76, 77]. Despite their heterogeneity, these examples illustrate a pattern wherein disorders involving DNA repair deficits manifest with diffuse neuropsychological impairment. This underscores the importance of considering cognitive and developmental sequelae in clinical evaluations of affected individuals.

There are limitations to this report. As a single-subject case report, these findings cannot be generalized to all FA patients. Cognitive phenotypes in FA may vary, and this patient may represent the more severe end of the cognitive spectrum. Second, there are multiple confounding factors in this patient's history that complicate interpretation of her neuropsychological results. She has a longstanding history of alcohol use disorder and mood disturbance, each of which can independently contribute to cognitive impairment [78–81]. It will be important in future studies to determine to what extent cognitive impairments in FA are due to the neurobiological effects of FA itself, the neurotoxic and psychological effects of alcohol use and mood disorders, or the interaction of FA with acquired environmental and/or psychiatric dysfunction. Notably, mood disorders are commonly observed among individuals with FA [20, 21, 23]. As such, this patient's profile, including psychiatric distress, remains clinically relevant to the FA community and their treating providers.

This case study highlights the critical need for systematic research on cognition in FA. Large-scale studies should be undertaken to determine how common and severe neuropsychological impairments are in the broader FA population. For example, the International Fanconi Anemia Registry (IFAR), which has successfully collected clinical data on hundreds of FA patients [3, 5], could incorporate standardized cognitive assessments into its protocol. Adding cognitive and behavioral measures to such registries would help establish a baseline neuropsychological profile for FA and allow researchers to correlate cognitive outcomes with genetic subtypes, medical variables, and treatments. Longitudinal studies will be especially important to determine whether developmental cognitive deficits are stable or progressive.

Clinicians should be aware of the potential for significant cognitive impairment in FA and consider cognitive screenings or comprehensive neuropsychological evaluations as part of routine care. Early identification of neurocognitive strengths and weaknesses may help inform academic accommodations and interventions, as well as need for psychosocial supports. More widespread comprehensive understanding may also decrease instances of misdiagnosis or misattribution of symptoms (e.g., cerebral calcifications) seen in FA. Ultimately, integrating cognitive data into FA research and care will fill a critical gap in the literature and identify need for interventions to improve quality of life for those living with FA.

## Figures and Tables

**Table 1:** Neuropsychological measures administered and corresponding neuroanatomical regions.

Measure	Subtest	Domain(s) assessed	Neuroanatomical correlates	Reference(s)
Advanced clinical systems	Test of premorbid functioning	Premorbid functioning	Left anterior temporal lobe; left occipitotemporal fusiform cortex	[26, 27]

Wechsler Adult Intelligence Scale-IV	Vocabulary	Vocabulary knowledge	Left inferior frontal gyrus; left anterior temporal lobe	[28–31]
	Block design	Visuoconstruction (timed)	Right inferior parietal lobe; right dorsolateral prefrontal cortex	
	Digit span	Basic auditory attention; working memory	Left supramarginal gyrus; left dorsolateral prefrontal cortex	
	Symbol search	Processing speed	Bilateral occipitoparietal visual association cortices; dorsolateral prefrontal cortex	
	Coding	Processing speed	Left frontoparietal network; primary motor cortex	

Grip strength test	—	Grip strength	Contralateral primary motor cortex; supplementary and premotor areas	[32–34]

Grooved Pegboard Test	—	Fine motor dexterity and speed	Primary motor cortex; cerebellum	[32, 34]

Boston Naming Test	—	Confrontation naming	Left anterior temporal lobe; left inferior frontal gyrus	[32, 35]

Verbal fluency	F/A/S	Lexical fluency	Left inferior frontal gyrus; left pre-supplementary motor area and caudate nucleus	[32, 36, 37]
	Animals	Semantic fluency	Left lateral temporal cortex; left dorsolateral prefrontal cortex	

Brief Visuospatial Memory Test	—	Visual learning and memory; visuoconstruction (untimed)	Right hippocampus/medial temporal lobe; right parietal-occipital cortex	[38, 39]

Rey Auditory Verbal Learning Test	—	Verbal learning and memory (list)	Left hippocampus/medial temporal lobe; frontoparietal network	[40–43]

Weschler Memory Scales-IV	Logical memory	Verbal learning and memory (story)	Left hippocampus/medial temporal lobe; medial prefrontal cortex	[44–46]

Trail Making Test Parts A & B	—	Processing speed; response inhibition	Intraparietal sulcus; visual association cortex; anterior cingulate cortex; dorsolateral prefrontal cortex	[32, 47]

Stroop Color and Word Test	Color/word reading	Processing speed	Left inferior frontal and lateral temporal cortices	[48–50]
	Color/word interference	Response inhibition	Anterior cingulate cortex; dorsolateral prefrontal cortex	

Wisconsin Card Sorting Test	—	Novel problem solving	Dorsolateral prefrontal cortex; caudate nucleus; parietal cortex	[51–53]

**Table 2:** Test performance by cognitive domain.

Domain		*T* score	Exceptionally low ≤ 2	Below average 2–8	Low average 9–24	Average 25–74	High average 75–90	Above average 91–97	Exceptionally high ≥ 98
Global functioning	Estimated premorbid (word reading)	*T* = 38			X				
	Estimated FSIQ	*T* = 30	X						

Language	Vocabulary	*T* = 33		X					
	Confrontation naming	*T* = 44				X			
	Semantic fluency	*T* = 48				X			
	Lexical fluency	*T* = 32		X					

Motor	Strength (dominant)	*T* = 25	X						
	Strength (nondominant)	*T* = 25	X						
	Speed/Dexterity (dominant)	*T* = 29	X						
	Speed/Dexterity (nondominant)	D/C	X						

Visuospatial	Visuoconstruction (untimed)	WNL				X			
	Visuoconstruction (timed)	*T* = 37			X				

Learning and memory	Verbal learning (list)	*T* = 26	X						
	Verbal recall (list)	*T* = 28	X						
	Verbal recognition (list)	*T* = 40			X				
	Verbal learning (story)	*T* = 30	X						
	Verbal recall (story)	*T* = 23	X						
	Verbal recognition (story)	—		X					
	Visual learning	*T* < 20	X						
	Visual recall	*T* < 20	X						
	Visual recognition	—		X					

Attention and executive functions	Basic auditory attention	*T* = 40		X					
	Auditory working memory	*T* = 37			X				
	Processing speed (coding; symbol search, trails A)	*T* = 30; *T* = 33; *T* = 33		X					
	Response inhibition	*T* = 28				X			
	Novel problem solving	*T* < 20	X						

*Note:* All scores are converted to T-scores; T-scores are standard scores with a mean of 50 and a standard deviation (SD) of 10. Performance descriptors and associated percentiles are reported as per the American Academy of Clinical Neuropsychology recommendations on uniform labeling of performance test scores [54]. Abbreviations: D/C = discontinued; WNL = within normal limits.

## Data Availability

The data that support the findings of this study are available on request from the corresponding author. The data are not publicly available due to privacy or ethical restrictions.
